# Clinical Profile and Length of Hospital Stay in a Sample of Psychogeriatric Patients Referred to Consultation Liaison Psychiatric Unit

**DOI:** 10.3390/medicina57030256

**Published:** 2021-03-11

**Authors:** Bernardo J. Barra, Luis F. Varela, José R. Maldonado, Pilar Calvo, Anna Bastidas, Roberto Sánchez, Luis Pintor

**Affiliations:** 1 Department of Psychiatry, Hospital Clinic i Provincial of Barcelona, University of Barcelona, Casanova Street, 143, 08036 Barcelona, Spain; bastidas@clinic.cat (A.B.); lpiNTOR@clinic.cat (L.P.); 2 Department of Psychiatry, Medicine School, Andrés Bello University of Santiago (UNAB), Santiago 8320000, Chile; l.varela@uandresbello.edu; 3 Psychiatry and Mental Health Service, CRS El Pino Hospital, South Metropolitan Health Service, Santiago 8320000, Chile; 4 Department of Psychiatry and Behavioral Sciences, Stanford University Medical Center, Stanford, CA 94305, USA; jrm@stanford.edu; 5 Medicine School, University of Chile, Santiago 8320000, Chile; PILUCALVO@GMAIL.COM; 6 Department of Psychiatry, Institute of Neuropsychiatry and Addictions, Parc de Salut Mar, 143, 08036 Barcelona, Spain; RSanchezGonzalez@parcdesalutmar.cat; 7 Institute of Biomedical Research August Pi i Sunyer (IDIBAPS), University of Barcelona, 143, 08036 Barcelona, Spain

**Keywords:** psychogeriatric, consultation liaison psychiatry, length of hospital stay, inpatients, youngest-old, oldest-old

## Abstract

*Background and objectives*: There has been a recent increase in older patients admitted to general hospitals. A significant percentage of hospitalized older patients are ≥75 years old, which differ from the patients aged 65 to 74 years old in terms of functional status at patient discharge. This study aims to compare sociodemographic, clinical features, and factors associated with length of hospital stay in youngest-old and oldest-old populations of inpatients referred to the consultation liaison psychiatry unit. *Material and methods*: This is an observational, cross-sectional, retrospective, and comparative study. We obtained data from a sample of 1017 patients (≥65 years) admitted to a general hospital and referred from different services (medicine, surgery, etc.) to the consultation liaison psychiatry unit. The sample was divided into two groups of patients: youngest-old (65–74 years) and oldest-old (≥75 years). Psychiatric evaluations were performed while the patients were on wards at the hospital. Psychopharmacs were started as needed. A comparative analysis was carried out and predictive factors related to length of hospital stay were calculated. *Results*: The reference rate to consultation liaison psychiatry unit was 1.45% of the total older patients hospitalized. Our study demonstrates differences between the groups of older people: the oldest-old group were mainly female (*p* < 0.001), had more previous psychiatric diagnoses (*p* < 0.001), physical disabilities (*p* = 0.02), and neurocognitive disorders (*p* < 0.001), they used more antipsychotics (*p* < 0.001), and more frequently had a discharge disposition to a nursing home (*p* = 0.036). The presence of physical disability (*beta* = 0.07, *p* < 0.001) and logtime to referral to consultation liaison psychiatry unit (*beta* = 0.58, *p* < 0.001) were associated with increased length of hospital stay. *Conclusions*: Youngest-old and oldest-old people should be considered as two different types of patients when we consider clinical features. The time to referral to consultation liaison psychiatry unit seems to be a relevant factor associated with length of hospital stay.

## 1. Introduction

There has been a recent increase in older patients admitted to general hospitals [1,2]. This increase has resulted in an increase in hospitalization periods, health costs, and morbidity and mortality rates in this group of patients [3,4]. A significant percentage of these older people admitted to general hospitals are ≥75 years old and differ from the patients aged 65 to 74 years old [5] in terms of functional status at the patient discharge and higher hospitalization risk at the emergency department [6,7]. These groups of older people admitted to general hospitals show psychiatric comorbidities in a range of 50–60%, implying a prevalence three to four times higher than that of those who live in the community [8]. The most prevalent psychiatric disorders in hospitalized older patients are delirium (61%), depression (53%), and dementia (40%) [9], which have been associated with high mortality [10,11]. 

Consultation liaison psychiatry (CLP) is the subspecialty of psychiatry concerned with medically and surgically ill patients who present psychiatric symptoms in a general hospital setting [12]. The referral rate of these patients to CLP units ranges between 0.72% to 6% [13]. Recently, there has been an increase in the reference rates to CLP units of people aged ≥65 years, as observed by Schellhorn [14]. Evaluation by CLP units have an important role, as there is evidence that CLP units are cost-effective and reduce the length of hospital stay when involved early; on the other hand, late time to referral means more prolonged length of hospital stay [15,16].

The objective of the present study is to evaluate sociodemographic and clinical features and factors associated with length of hospital stay in youngest-old (65–74 years old) and oldest-old (≥75 years old) populations admitted to a general hospital and referred to a CLP unit.

## 2. Material and Methods

### 2.1. Design

This is an observational, cross-sectional, retrospective, and comparative study carried out between 1 January 2016 and 31 December 2018 which gathered all the cases admitted to our unit from 2007 to 2014. The results are reported according to the STROBE statement [17,18].

### 2.2. Patients

**Figure 1:**
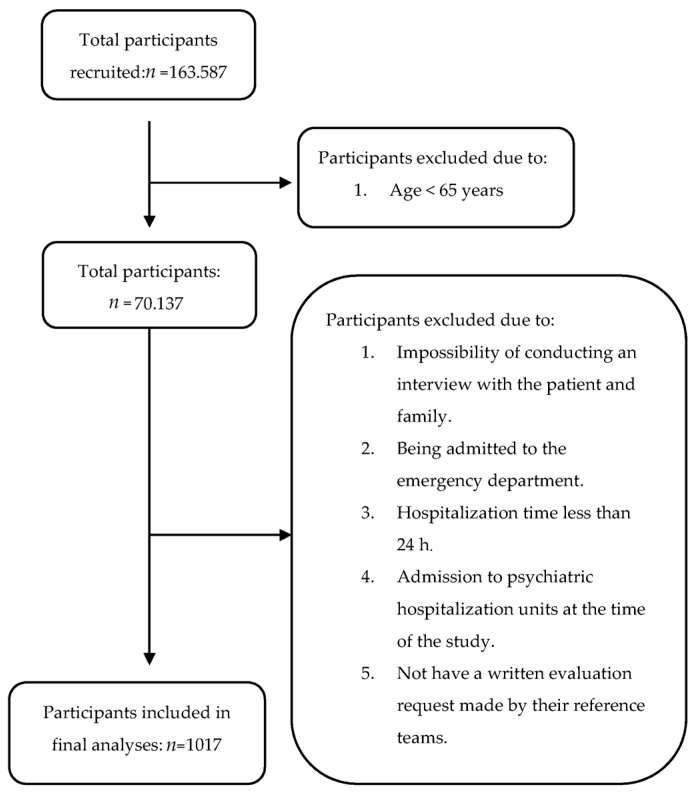
Study flow diagram.

Participants were recruited from the Clinic Hospital of Barcelona (CHB), which is a tertiary facility with 819 beds and a catchment area of 540,000 inhabitants within the metropolitan area of Barcelona. Participants met the study inclusion criteria if they were 65 years and older and were referred to the CLP unit from the different services medical/surgical of the CHB. The requests were made when the physicians detected a patient with psychiatric pathology. The sample for the analyses in this study was n = 1017 participants (Figure 1; Study Flow Diagram). The patients were classified into two groups, youngest-old (65–74 years old) and oldest-old (≥75 years old), following the division used in the study by Tadros et al. [19].

### 2.3. Data Sources and Procedure

The referrals were received by the CLP unit through the hospital intranet which delivered sociodemographic variables and clinical characteristics of the sample, including age, sex, and diagnoses of somatization according to the International Classification of Diseases (ICD-10) [20]. 

The request from the referring department contained the following variables: date, reference sources (medical specialties), reason for the referral, and brief medical history of the patient.

The initial interview was conducted by unit psychiatrists, and the anamnesis data were obtained from patients, family members, caregivers, reference physicians, and nurses.

The psychiatric diagnoses were made following the DSM-5 criteria [21].

The data of the patient’s follow-up during the hospital episode, such as psychopharmacological intervention, number of visits, length of hospital stay (LOS), and destination after discharge, were obtained by the psychiatrists and the unit nurse. All unit staff were trained in accordance with European guidelines, and all the cases they evaluated were reviewed by a board-certified faculty psychiatrist [22,23].

The data obtained were subsequently downloaded to the ACCESS software (Microsoft package), where they were stored according to the proposals of the European Consultation/Liaison Workgroup (ECLW) for standardized data collection [24].

### 2.4. Statistical Analyses

The statistical program IBM SPSS statistics 23 (IBM Corp., Armonk, NY, USA) for Microsoft Office 2013 was used. The comparative analysis between the youngest-old and the oldest-old population was carried out using the Pearson’s chi-squared test. Differences between groups regarding LOS and Time to Referral to consultation liaison psychiatry unit were analyzed with the Mann–Whitney U Test, taking into account their positively skewed distribution. For this same reason, logarithmic transformation was applied to both variables to obtain a logLOS and logTime to referral CLP unit in line with methods applied in the literature by Lyons et al. [25]. 

To determine possible associations between sociodemographic and clinical variables and LOS, a hierarchical multiple regression was conducted with logLOS defined as the dependent variable. Coefficients were interpreted as suggested by Benoit [26]. 

Our study was presented to Hospital Clinical Research Ethics Committee to obtain their approval to carry out the clinical study (Reg. HCB/20L6/0342 project identification code, date: 4 August 2016). All procedures followed the ethical principles for medical research established in the Declaration of Helsinki [27].

## 3. Results

The study population included a total of 1017 patients. During the study period, 163.587 patients were admitted to the Clinic Hospital of Barcelona (CHB), from which 70.137 (43%) were ≥65 years old. The reference rate to the CLP unit was 1.45% of the total number of hospitalized older patients. On average, the patients in the sample were 75.73 ± 6.5 years old. 

The socio-demographic characteristics are shown in Table 1. 

The oldest-old group had a higher proportion of women than men and presented with significantly more previous psychiatric diagnoses than the youngest-old. The oldest-old also displayed higher rates of physical disability, and therefore needed assistance more frequently than the youngest-old. These results are consistent with previous evidence and compatible with what is reasonable from the clinical point of view. Table 2 shows the characteristics of the references. 

The majority of referrals (59%) came from medical services. Both physicians and psychiatrists found more neurocognitive disorders in the oldest-old, and less substance dependence in the youngest-old group. Mood (37%) and neurocognitive disorders (31%) were the most frequent diagnosis referred by physicians to the CLP unit. The prevalence of diagnosed neurocognitive disorders was higher when the diagnosis was done by CLP unit psychiatrists than those made by the reference team. Comparing these disorders, non-psychiatrists diagnosed more mood disorders, while psychiatrists performed more diagnoses of neurocognitive disorders.

Table 3 shows the interventions used by CLP unit psychiatrists. Medication was prescribed to 83% of the total sample. The most prescribed drugs were antipsychotics (42%), followed by antidepressants (30%). Oldest-old patients were prescribed antipsychotics (245 out of 499) significantly more frequently than youngest-old patients (184 out of 518). 

Youngest-old patients were prescribed benzodiazepine medications two times more often (68 out of 518) than oldest-old patients (33 out of 499). The vast majority of our sample was visited between one and three times (81%). Hospitalized geriatric patients were often discharged to go home (82%), with youngest-old patients more frequently discharged to their homes than the oldest-old.

The median LOS for the whole sample was 16 days (Interquartile range (IQR) = 9–32 days). The median LOS for younger-old was 18 days (IQR = 9–37 days) and for oldest-old was 15 days (IQR = 9–28 days). There were no statistically significant differences between both groups (U = 28,810.5, *p* = 0.064). The median time to referral to CLP unit for the whole sample was six days (IQR = 3–14 days). Both groups showed those same values and therefore there were no statistically significant differences in time to referral to CLP unit (U = 50,251, *p* = 0.67).

Table 4 shows the results of the hierarchical multiple regression model in which logLOS was included as the outcome variable. Gender and geriatric group were entered at step 1, explaining 1.2% of the variance in logLOS. In step 2, dichotomic variables, history of previous psychiatric diagnosis, and physical disability were included. This model explained 9% of the variance in logLOS. 

In the final model, the variable “logTime to referral Consultation Liaison Psychiatry Service (days)” was included. This model was statistically significant (*F* (5, 483) = 112.8, *p* < 0.001) and explained 53% of the variance in logLOS. Belonging to the oldest-old group (*beta* = −0.06, *p* = 0.01) (6% decrease) and having history of previous psychiatric diagnosis (*beta* = −0.06, *p* = 0.03) (6% decrease) were associated with decreases in LOS. On the other hand, physical disability (*beta* = 0.07, *p* < 0.001) (7% increase) and log time to referral to CLP unit (*beta* = 0.58, *p* < 0.001) (for every 10% increase in time to referral to CLP unit, LOS increases by 5.7%) were associated with increased LOS. 

## 4. Discussion

The main finding of our study is the association between a longer delay in time to referral of elderly patients with some psychiatric pathology to the consultation liaison psychiatric unit by medical and surgical services, and an increase in the length of hospital stay. This finding is consistent with the results of Sockalingam et al. [28], which demonstrate that longer waiting time to referral to CLP unit leads to longer LOS, especially in elderly groups and patients with a diagnosis of acute delirium-type mental disorder (neurocognitive disorders). Some studies associate this delay with difficulties that non-psychiatric physicians experience in recognizing psychiatric pathology, especially delirium [29]. The longer hospital stay for these patients leads to an increase in morbidity, mortality, and institutionalization, as mentioned by the Royal College of Psychiatrists in guidelines for the development of a CLP unit for older people [30]. Prolonged hospital stays are a major challenge for health systems and present high associated economic costs. Research has confirmed how effective CLP unit clinical actions may help improve outcome indicators of health care, i.e., quality of life and disability of patients, length of hospital stay, and health costs [31]. A rapid and effective participation of the liaison psychiatrist is therefore relevant and is aligned with the proactive model of care of a CLP unit; this participation also focuses on early psychiatric care in medical and surgical settings, which has been shown to decrease LOS [32]. 

Patients in our study with a previous psychiatric diagnosis had shorter hospitalization, an outcome that contrasted with the study by Lewis et al. [33]. These authors point out that one of the factors for delays in hospital discharge is the presence of psychiatric problems. We believe that this could be due to a shorter time to referral to CLP unit by the non-psychiatry physician when they have knowledge of the psychiatric diagnosis prior to hospitalization.

Surprisingly, and contrary to the trend found in the literature [34], oldest-old patients in our sample had shorter hospital stays than younger patients. A similar finding was made by Chung et al. [35]. This difference could be due to the methodology employed in our study, namely the separation of elderly adults by age into subgroups to allow for more specific results. Another explanation could be related to the higher presence of neurocognitive disorders, such as delirium, in the oldest-old patients; this might reduce the time to referral by non-psychiatrist physicians to the CLP unit, which in turn could lead to an earlier evaluation and management from the CLP unit, and a reduction in length of hospital stay. We also observed a low rate of referral of elderly patients (1.45%) from the different hospital services to the CLP unit, which contrasts with the higher figures expected for this age group in recent years (1.99–3.95%), as can be seen in the study by Anderson et al. [36]. This could be related to the patient’s preferences, stigma, poor relationship with the psychiatrist, negative attitude towards CLP, or problems in the recognition of psychiatric diseases by non-psychiatrists, as indicated in the study carried out by Chen et al. [37]. 

The youngest-old population was different from the oldest-old population in this study. Oldest-old demonstrated a greater referral rate to CLP unit by surgical services, and a significantly higher percentage of neurocognitive disorders compared to the younger population; this observation is consistent with findings by Schellhorn et al. [14]. These features may be related to the high association between severe surgical and medical pathology and the development of delirium in oldest-old patients. The high rate of referrals from surgical services to CLP unit in the general sample (328 out of 1017) (32%) contrasts with other studies such as Yamada et al. [38], where the referral rate of elderly patients from the surgery service did not exceed 22%.

The more frequent use of antipsychotics in the oldest-old group could be explained by the greater prevalence of neurocognitive disorders and behavioral disturbances frequently associated with delirium and dementia [39], which are usually treated with antipsychotics in the routine clinical practice [40]. Another explanation could be the correct evaluation of pharmacological risks and benefits of antipsychotics that, despite side effects at the metabolic level, have a higher safety profile than benzodiazepines when used for sedation in these patients [41]. Although antipsychotics are frequently used in clinical practice, it is important to note that their use has limited efficacy in reducing the severity and resolution of delirium symptoms compared to non-antipsychotic drugs, as can be seen in the study by Burri et al. [42]. Furthermore, in patients with dementia the efficacy of antipsychotics on neuropsychiatric symptoms is also limited, in addition to the risk of serious side effects, such as cardiorespiratory arrest, stroke, falls, arrhythmias, extrapyramidal signs and mortality, especially in the patient group 65 years or older. The assessment of safety risks versus expected benefits should be individualized when prescribing these drugs in this type of patient [43,44].

As in previous research [45,46], most of our patients received between one and three visits from the CLP unit, and the number of visits was significantly higher in the oldest-old group. This observation could be related to a greater severity of psychiatric pathology (higher prevalence of neurocognitive disorders, e.g., delirium) in this group. 

For the post-hospital discharge referral, the oldest-old group was more frequently referred to a nursing home. This coincides with the findings of O’Sullivan et al. [47], who suggested the association between a neurocognitive disorder (delirium type or dementia) during hospitalization and adverse outcomes such as longer length of hospital stay and greater functional decline; this association reflected a loss of independence in daily living activities and prolonged cognitive impairment. These outcomes lead to an increased need for specialized care for this population. 

### Limitations

There are several limitations to this study. First, the study is cross-sectional, which may allow biases. Another limitation is the lack of previous research studies on the subject. The study was performed in a university-based hospital in an urban area, which does not allow the results to be generalized. 

No screening instruments such as scales were used to perform psychiatric diagnoses. The follow-up of the patients was only performed during hospitalization. The sample was selected by non-psychiatrists. It was not an active search for all hospitalized elderly patients, which would have given us a less-biased sample. We have not collected or considered aspects of medical pathology or the pharmacological approach beyond psychoactive drugs. Another limitation is that some of our patients with diagnoses of acute delirium and severe dementia could not communicate in an orderly manner, which may have generated some inaccuracies. Our study is retrospective, naturalistic, and of clinical practice, which can generate more inaccuracies without a protocolized follow-up of a fixed number of visits. Another of our limitations is the absence of a control group, which would allow us to obtain the association between time to referral and total LOS with greater certainty. 

## 5. Conclusions

The increase in the occupation of hospital beds by older people in recent years has generated more referrals from this age group to CLP units. Youngest-old and oldest-old people should be considered as two different types of patients when we consider their clinical features. The time to referral to CLP units seems to be a relevant factor associated with length of hospital stay in these age groups. CLP units will need to place more emphasis on older people to meet this demand, and have professionals trained in geriatric psychiatry to address the needs of this group. In addition, we believe it is necessary to carry out randomized clinical trials, which make it possible to deepen the association between a longer delay in the time to referral by non-psychiatrists from hospital services to the CLP units, and the longer length of hospital stay. These additional studies will also facilitate a better understanding of the different clinical profiles among elderly patients, which would allow us to carry out more specific interventions according to their needs.

## Figures and Tables

**Table 1:** Comparison sociodemographic characteristics of the sample.

		Youngest-Old *N* = 518	Oldest-Old *N* = 499	Total *N* = 1017	*p* Value
Gender	Male	297 (59%)	206 (41%)	503 (100%)	*p* < 0.001 **
	Female	221 (43%)	293 (57%)	514 (100%)	*p* < 0.001 **
Medical Diagnosis	General Medicine	183 (55%)	152 (45%)	335 (100%)	NS
	Medical Subspecialties ^†^	203 (48%)	216 (52%)	419 (100%)	NS
	Neurology	48 (62%)	30 (38%)	78 (100%)	NS
	Psychiatry	29 (59%)	20 (41%)	49 (100%)	NS
	Surgery ^‡^	55 (40%)	81 (60%)	136 (100%)	*p* = 0.08
Previous Psychiatric Diagnosis	Yes	227 (45%)	277 (55%)	504 (100%)	*p* < 0.001 **
	No	291 (57%)	222 (43%)	513 (100%)	*p* < 0.001 **
Physical Disability	Autonomous	293 (56%)	234 (44%)	527 (100%)	*p* = 0.02 *
	Needs Assistance	225 (46%)	265 (54%)	490 (100%)	*p* = 0.02 *

Abbreviations: NS = Not Significant. † Includes: Cardiology, Hematology/Oncology, Nephrology/Urology Services. ‡ Includes: Surgery and Trauma Services. Note: *p* values were calculated using the Pearson’s chi-square test. * *p* < 0.05; ** *p* < 0.01.

**Table 2:** Characteristics of the references: Source of referral, axis I diagnosis (service referral, consultation liaison psychiatry (CLP) unit). Comparative analysis.

		Youngest-Old *N* = 518	Oldest-Old *N* = 499	Total *N* = 1017	*p* Value
Referring Service	General Medicine	156 (53%)	141 (47%)	297 (100%)	NS
	Medical Subspecialties ^†^	184 (60%)	124 (40%)	308 (100%)	*p* < 0.001 **
	Surgery ^‡^	124 (39%)	204 (62%)	328 (100%)	*p* < 0.001 **
	Neurology	54 (64%)	30 (36%)	84 (100%)	*p* = 0.01 *
Psychiatric Diagnosis Referring Service	Anxiety Disorder	60 (61%)	38 (39%)	98 (100%)	*p* = 0.032 *
	Mood Disorder	187 (50%)	185 (50%)	372 (100%)	NS
	Neurocognitive Disorder ^§^	124 (40%)	187 (60%)	311 (100%)	*p* < 0.001 **
	Substance Dependence	57 (83%)	12 (17%)	69 (100%)	*p* < 0.001 **
	Psychosis	13 (43%)	17 (57%)	30 (100%)	NS
	Personality Disorder	6 (50%)	6 (50%)	12 (100%)	NS
	Adaptive Disorder	55 (60%)	36 (40%)	91 (100%)	NS
	Suicide Attempt	16 (47%)	18 (53%)	34 (100%)	NS
Psychiatric Diagnosis CLP Unit	Anxiety Disorder	19 (53%)	17 (47%)	36 (100%)	NS
	Mood Disorder	101 (61%)	65 (39%)	166 (100%)	*p* = 0.005 **
	Neurocognitive Disorder	182 (41%)	264 (60%)	446 (100%)	*p* < 0.001 **
	Substance Dependence	56 (82%)	12 (18%)	68 (100%)	*p* < 0.001 **
	Psychosis	23 (52%)	21 (48%)	44 (100%)	NS
	Adaptive Disorder	98 (54%)	85 (46%)	183 (100%)	NS
	Personality/Somatoform Disorder	13 (62%)	8 (38%)	21 (100%)	NS
	No Diagnosis	26 (49%)	27 (51%)	53 (100%)	NS

Abbreviations: NS, Not Significant; CLP, Consultation Liaison Psychiatry. Note: *p* values were calculated using the Pearson’s chi-squared test. * *p* < 0.05; ** *p* < 0.01. † Includes: Cardiology, Hematology/Oncology, Nephrology/Urology Services. ‡ Includes: Surgery and Trauma Services. § Includes: Delirium and Dementia.

**Table 3:** Comparison of central nervous system active medications, psychiatric follow-up of patients, and discharge disposition.

		Youngest-Old *N* = 518	Oldest-Old *N* = 499	Total *N* = 1017	*p*-Value
Pharmacological Prescription by CLP Unit	Antidepressant	166 (56%)	132 (44%)	298 (100%)	NS
	Antipsychotic	184 (43%)	245 (57%)	429 (100%)	*p* < 0.001 **
	Mood Stabilizer	12 (63%)	7 (37%)	19 (100%)	NS
	Benzodiazepine	68 (67%)	33 (33%)	101 (100%)	*p* = 0.001 **
	No Prescription	88 (52%)	82 (48%)	170 (100%)	NS
Number of Visits by CLP Unit	1	196 (58%)	139 (42%)	335 (100%)	*p* = 0.001 **
	2–3	226 (46%)	264 (54%)	490 (100%)	*p* = 0.003 **
	4–7	74 (49%)	78 (51%)	152 (100%)	NS
	>7	22 (55%)	18 (45%)	40 (100%)	NS
Discharge Disposition	Nursing Home	56 (42%)	76 (58%)	132 (100%)	*p* = 0.036 *
	Home	438 (53%)	396 (47%)	834 (100%)	*p* = 0.031 *
	Death	16 (44%)	20 (56%)	36 (100%)	NS
	Others	8 (53%)	7 (47%)	15 (100%)	NS

Abbreviations: NS, Not significant; CLP, Consultation Liaison Psychiatry. Note: *p* values were calculated using the Pearson’s chi-squared test. * *p* < 0.05; ** *p* < 0.01.

**Table 4:** Linear model of predictors of the length of hospital stay.

Step		b (95% CI)	SE	P
1	Gender	−0.03 (−0.11, 0.04)	0.04	0.4
	Geriatric Group ^†^	−0.09 (−0.16, −0.01)	0.04	0.02 *
2	Gender	−0.03 (−0.10, 0.04)	0.04	0.3
	Geriatric Group ^†^	−0.11 (−0.18, −0.04)	0.04	<0.001 **
	History of Previous Psychiatric Diagnosis ^‡^	−0.16 (−0.23, −0.09)	0.04	<0.001 **
	Physical Disability ^§^	0.15 (0.08, 0.22)	0.04	<0.001 **
3	Gender	−0.02 (−0.07, 0.03)	0.03	0.5
	Geriatric Group ^†^	−0.06 (−0.11, −0.01)	0.03	0.01 *
	History of Previous Psychiatric Diagnosis ^‡^	−0.06 (−0.11, −0.01)	0.03	0.03 *
	Physical Disability ^§^	0.07 (0.02, 0.12)	0.03	<0.001 **
	Log Time to Consultation-Liaison Psychiatry Service (days)	0.58 (0.53, 0.63)	0.03	<0.001 **

Abbreviations: b, beta; SE, Standard error; P, *p*-value. * *p* < 0.05; ** *p* < 0.01. ^†^ Coded 0 = Youngest-old, 1 = Oldest-old. ^‡^ Coded 0 = no, 1 = yes. ^§^ Coded 0 = no, 1 = yes.
